# The Impact of the Duration of Adjuvant Chemotherapy on Survival in Patients with Epithelial Ovarian Cancer – A Retrospective Study

**DOI:** 10.1371/journal.pone.0169272

**Published:** 2017-01-06

**Authors:** Veronika Seebacher, Alexander Reinthaller, Heinz Koelbl, Nicole Concin, Regina Nehoda, Stephan Polterauer

**Affiliations:** 1 Department for Gynecology and Gynecologic Oncology, Gynecologic Cancer Unit, Comprehensive Cancer Centre, Medical University of Vienna, Vienna, Austria; 2 Karl Landsteiner Institute for General Gynecology and Experimental Gynecologic Oncology, Vienna, Austria; 3 Department for Gynecology and Obstetrics, Medical University of Innsbruck, Innsbruck, Austria; Taipei Medical University, TAIWAN

## Abstract

**Objective:**

The aim of the present study was to investigate the prognostic role of the duration of adjuvant chemotherapy in patients with epithelial ovarian, fallopian tube and primary peritoneal cancer (EOC).

**Materials and Methods:**

Within the present study we retrospectively evaluated the data of 165 consecutive patients with EOC treated with primary surgery followed by six completed cycles of platinum-taxan based intravenous adjuvant chemotherapy. Medians of total duration of chemotherapy were compared with clinical-pathological parameters. Patients were stratified into four risk groups according to the delay in days of total duration of chemotherapy, and univariate and multivariable survival analyses were performed.

**Results:**

The median duration of six completed cycles of chemotherapy comprised 113 days (IQR 107–124 days). Uni- and multivariable survival analyses revealed a delay of total duration of chemotherapy of at least 9 days to be associated with progression-free (PFS), cancer-specific (CSS) and overall survival (OS). Hazard ratios (HR), confidence intervals (95% CI) and p-values for PFS, CSS and OS due to delay of chemo-duration were 2.9 (1.6–5.4; *p = 0*.*001*), 2.9 (1.3–6.2; *p = 0*.*008*) and 2.6 (1.3–5.4; *p = 0*.*008*), respectively.

Prolonged total chemo-duration was associated with the amount of postoperative residual disease *(p = 0*.*001)* and the patients’ age *(p = 0*.*03)*.

**Conclusion:**

The present study suggests a prolonged duration of adjuvant chemotherapy after primary surgery to adversely affect PFS, CSS and OS in patients with EOC. Yet larger studies are required to validate our results.

## Introduction

Epithelial ovarian cancer (EOC) is the second most common gynecological cancer, and, in 2012, was accountable for the deaths of 151.917 women worldwide [1]. As EOC causes few symptoms in its early stage, in approximately 75% of the patients disease has spread throughout the peritoneal cavity already at the time of diagnosis. Therapeutic success is dependent on two essential pillars: the achievement of optimal cytoreductive surgery (CRS) and the response to adjuvant chemotherapy [2]. Over the past decades the combination of a platinum and taxan based regimen has proved best response rates [3, 4]. A consolidation therapy after six cycles did not show improvement of survival so far [5]. Even after optimal CRS and complete response to adjuvant chemotherapy approximately two thirds of the patients experience progression of disease within 18 to 24 months [3].

Age at diagnosis, tumor stage and the amount of residual disease after CRS are widely recognized as prognostic parameters influencing survival [6]. In cervical and anal cancer the duration of chemotherapy was reported to have a decisive effect on the patients’ survival [7, 8]. In EOC data on the association between duration of chemotherapy and survival outcome are partially inconsistent. The role of relative dose intensity (RDI), the ratio between planned and received dose intensity, as prognostic marker for survival was investigated by several authors. An older study thereby could not find an association between RDI and survival [9]. Yet, more recently, RDI of less than 70% and 85% was reported to be associated with shorter progression-free survival (PFS) by two studies [10, 11], and in addition with shorter overall survival by a third study [12]. Only one study reported on the investigation of the role of a delay of the duration of chemotherapy separately from dose reduction. This study found no association between delay of chemo-duration or dose reduction and the patients’ survival [13].

Otherwise the impact of the duration of adjuvant chemotherapy on the course of EOC has not been sufficiently investigated. We performed this study to evaluate the prognostic significance of the duration of adjuvant chemotherapy on survival in patients with EOC.

## Material and Methods

### Patients

By using the electronic gynecologic oncology registry we retrospectively identified all patients with pathologically confirmed epithelial ovarian, fallopian tube, and primary peritoneal cancer (EOC) treated at the Medical University of Vienna between 1996 and 2010. Patients’ records were reviewed to identify all patients who were initially treated with CRS followed by six completed cycles of adjuvant intravenous chemotherapy combining carbo-/cisplatin with pacli-/docetaxel. Patients were excluded from analyses if they had non-epithelial or borderline histology, if they were treated by neoadjuvant or intraperitoneal chemotherapy, if they received more or less than six cycles of chemotherapy, and if regimens other than a platinum and taxan combination were used. Clinical and pathological data were extracted from the respective electronic and paper-based patients’ records.

### Clinical management

All included patients were treated by upfront CRS and staged according to 1988 FIGO (International Federation of Gynecologists and Obstetricians) guidelines [14]. In all patients, total hysterectomy, bilateral salpingo-oophorectomy and omentectomy were performed. In FIGO stages Ia to Ic pelvic and paraaortic lymph node staging were performed. In FIGO stages II and above, only suspicious lymph nodes were removed. Furthermore, in early stages peritoneal biopsies were taken. In advanced stages cytoreductive surgery was done as appropriate with the goal to remove all visible disease. All patients received six cycles of a 21 days regimen of adjuvant intravenous chemotherapy combining carbo-/cisplatin with pacli-/docetaxel. The duration of chemotherapy was measured from the day of first given dose to the day of last given dose. A total chemo-duration of 15 weeks (5 x 21 days) was thereby considered as optimal time frame.

After completion of treatment, follow-up visits including clinical examination and serological tumor marker evaluation were scheduled four times a year for years one to three, twice a year for years four to six, and once a year for years seven to ten. Pelvic ultrasound examination and computed tomography of thorax and abdomen were performed once a year or when clinically indicated. If patients did not present for scheduled follow-up visits administrative personnel or nurses contacted them.

### Statistical analysis

Values are given as means (standard deviation [SD]) or medians (interquartile range [IQR]) as appropriate.

Independent Samples Kruskal-Wallis test was used to assess the association between the median chemo-duration and clinico-pathological parameters.

For survival analyses we assigned patients to risk groups according to the duration of delay of total chemo-duration. No or up to 2 days delay was considered as “optimal reference”. Additional groups were delays of total chemo-duration of 3 to 8, 9 to 19, and more than 19 days. These groups were in alignment with the quartiles of distribution of chemo-duration within the study population.

Survival probabilities were calculated by the product limit method of Kaplan and Meier. Differences between groups were tested using the log-rank test. The results were analyzed for the endpoints of progression-free (PFS), cancer-specific (CSS), and overall survival (OS). For PFS events were defined as the date of progression. For CSS events were defined as cancer-related death. For OS events were defined as cancer-related death and death due to any cause. Patients who were still alive were censored with the date of last follow-up. Multivariable Cox regression models were performed to adjust the effects of the delay of chemo-duration to the FIGO tumor stage (FIGO IV vs. III vs. II vs. I), the histological grading (G3 vs. G2 vs. G1), and the patients’ median age (≤ 58 vs. > 58 years).

Results of univariate and multivariable survival analyses are given as p-value (hazard-ratio [HR] and 95% confidence interval [95%CI]). P-values <0.05 were considered statistically significant. We used the statistical software SPSS 16.0 for Mac (SPSS 16.0.1, SPSS Inc., Chicago, IL) for statistical analysis.

### Institutional review board

The present study was approved by the institutional review board, the Ethics-Committee of the Medical University of Vienna (Project # 266/2010). Since this is a retrospective study informed consent was not requested by the institutional review board. The patient data was de-identified and handled in accordance with ethical standards of good scientific practice.

## Results

### Patients’ characteristics

**Fig 1:**
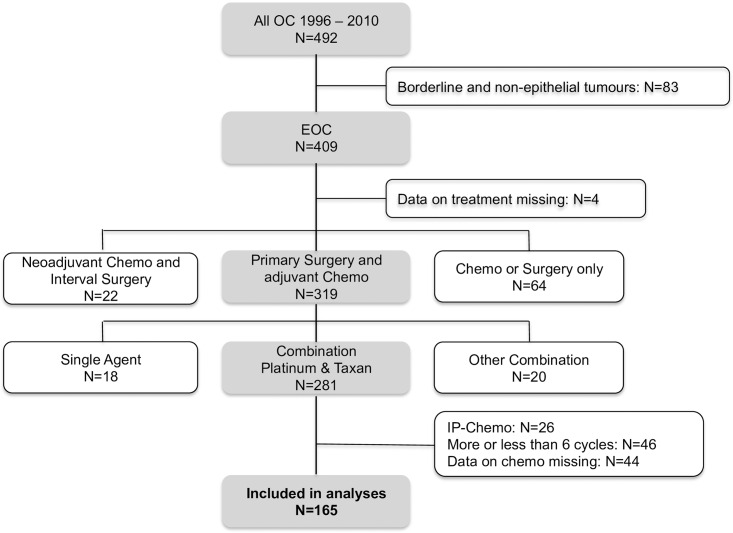
Flowchart. The process of inclusion and exclusion of patients in order to find the sample of patients included in the present study (= 165).

We could identify 492 patients who were treated for ovarian, fallopian tube and primary peritoneal cancer in the years 1996 to 2010 at the Medical University of Vienna, Austria. The flowchart in Fig 1 depicts reasons for excluding patients from the present study (Fig 1). A total of 165 patients were finally eligible and therefore included into the present analyses.

Pathological and treatment associated results are listed in Table 1. The patients’ median age was 58 years (IQR 49–64). The median duration of six completed cycles of chemotherapy (total chemo-duration) was 113 days (IQR 107–124 days). In 47 patients (28.5%) all six cycles could be administered with either no or only up to 2 days delay from the optimal time frame of 15 weeks. A delay of total chemo-duration of 3 to 8 days was noted in 38 patients (23%), of 9 to 19 days in 40 patients (24.2%), and of more than 19 days in 45 patients (24.2%). A delay of a chemo-cycle for at least one or ≥ 2 weeks was noted in 20 (12.1%) and 14 (8.4%) patients for cycle 2, in 14 (8.4%) and 9 (5.4%) for cycle 3, in 11 (6.7%) and 13 (7.8%) for cycle 4, in 14 (8.4%) and 6 (3.6%) for cycle 5, and in 15 (9.1%) and 21 (12.7%) for cycle 6, respectively. Reasons for delay of chemo cycles were given in 74 out of 137 cases of chemo-delay. These reasons comprised haematotoxicity (thrombocytopenia, neutropenia, neutropenic fever) in 45 (32.8%), impairment of wound healing in 6 (4.4%), surgical complications (ileus, bowel perforation, fistula) in 5 (3.6%), urinary tract infection in 2 (1.5%), nephrotoxicity in 2 (1.5%), liver toxicity in 1 (0.7%), venous thrombosis in 1 (0.7%), other medical conditions in 10 (7.2%), and patient wish in 2 cases (1.5%). For the residual 63 cases, organizational issues or patient wish are assumed, as medical conditions would have been annotated. In one patient the dose of carboplatin and paclitaxel was reduced to 75% for cycles 3 to 6. All other patients received the full dose.

**Table 1:** Patients’ characteristics (n = 165).

Parameter	Number (%)
Stage*	
FIGO Ia	6 (3.6)
FIGO Ib	1 (0.6)
FIGO Ic	27 (16.4)
FIGO IIa	5 (3.0)
FIGO IIb	8 (4.8)
FIGO IIc	5 (3.0)
FIGO IIIa	8 (4.8)
FIGO IIIb	14 (8.5)
FIGO IIIc	81 (49.2)
FIGO IV	10 (6.1)
**Postoperative residual disease**°	
< 5mm	74 (68.5)
5mm– 10mm	12 (11.1)
> 10mm	22 (20.4)
**Grade**	
G1	20 (12.1)
G2	42 (25.5)
G3	95 (57.6)
N/A	8 (4.8)
**Histological subtype**	
Serous papillary adenocarcinoma	92 (55.8)
Mucinous adenocarcinoma	8 (4.8)
Endometrioid adenocarcinoma	35 (21.3)
Clear cell adenocarcinoma	8 (4.8)
Undifferentiated adenocarcinoma	22 (13.3)
**Median time of follow-up in months (IQR)**	53.7 (29.5–85.5)
Number of patients with progressive disease	93 (56.4)
Median time to progression in months (IQR)	16.6 (12.3–26.9)
**Status at last observation**	
Disease-free	74 (44.8)
Stable disease	6 (3.6)
Progressive disease	12 (7.4)
Died from any cause	4 (2.4)
Died from ovarian cancer	69 (41.8)

N/A: data not available

*according to the 1988 FIGO staging system

°information on postoperative residual disease available in 108 patients (65.5%)

### Association between total chemo-duration and clinico-pathological parameters

Results of comparisons between median total chemo-duration and clinico-pathological parameters are given in Table 2. We could not show an association between prolonged total chemo-duration and FIGO tumor stage. Yet, prolonged total chemo-duration was associated with the amount of postoperative residual disease *(p<0*.*001)* and the patients’ age *(p = 0*.*03)*.

**Table 2:** Median duration of chemotherapy broken down by clinical and pathological parameters.

	Median (IQR) duration of chemotherapy in days*	P
**FIGO Stage**^#^		0.3
I	110.0 (106–120.5)	
II	112.5 (107–119.2)	
III	115 (108–127)	
IV	113 (104–126.2)	
**Postoperative residual disease**		<0.001
< 5 mm	111 (106–119.2)	
≥ 5 mm and < 10 mm	119.5 (110–144.5)	
≥ 10 mm	124 (114.7–131.7)	
**Age**		0.03
≤ 58 years	110 (106–120)	
> 58 years	115 (110–127)	

SD = standard deviation

^#^ FIGO (International Federation of Gynecology and Obstetrics) tumor stage

*Independent Samples Kruskal-Wallis Test

### Association between total chemo-duration and survival

**Fig 2:**
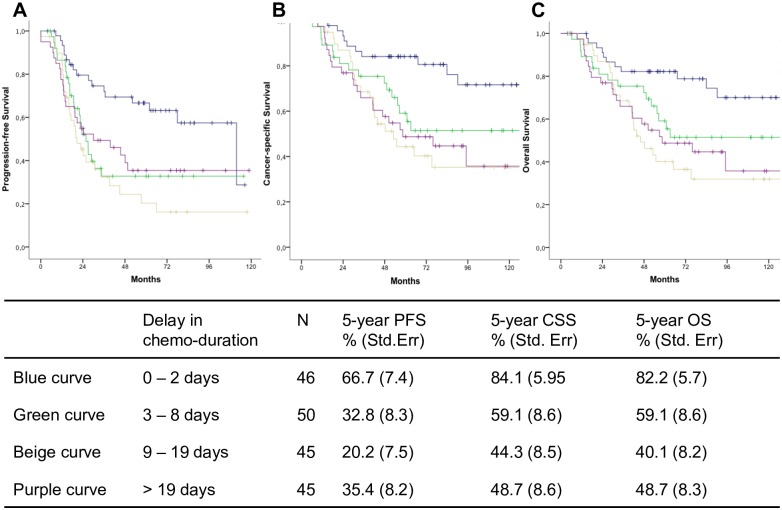
Survival curves. Kaplan Meier curves for patients stratified into risk groups according to the delay of total chemo-duration are given for progression-free (PFS), cancer-specific (CSS), and overall survival (OS) in figures A, B, and C, respectively. 5-year rates of PFS, CSS and OS for each risk group are given in the table below.

For survival analyses we assigned patients to the above defined risk groups according to the amount of delay of total chemo-duration. We compared survival outcome of patients in the three risk groups “3 to 8 days”, “9 to 19 days”, and “more than 19 days” to the group “0 to 2 days” delay as reference. A delay of total chemo-duration of 3–8 days was associated with shorter PFS in uni- and multivariable analyses, and with shorter CSS in uni-, but not multivariable analysis. A delay of 9 days or more was significantly associated with shorter PFS, CSS, and OS in univariate survival analyses. Furthermore, this effect remained significant after adjustment to the effects of other prognostic parameters, such as FIGO tumor stage, histological grading, and the patients’ age. Detailed results of uni- and multivariable survival analyses including hazard ratios, 95% confidence intervals, and p-values are given in Table 3. Furthermore, Kaplan Meier curves for PFS, CSS, and OS for patients stratified to the three risk groups are depicted in Fig 2.

**Table 3:** Hazard ratios of uni- and multivariable analyses for the association between delay of total chemo-duration and progression-free (PFS), cancer-specific (CSS), and overall survival (OS).

Delay in total chemo-duration	N	Progression free Survival				Cancer-specific Survival				Overall Survival			
		P	Unadjusted HR (95% CI)	P	Adjusted HR° (95% CI)	P	Unadjusted HR (95% CI)	P	Adjusted HR° (95% CI)	P	Unadjusted HR (95% CI)	P	Adjusted HR° (95% CI)
0–2 days	47		Ref		Ref		Ref		Ref		Ref		Ref
3–8 days	38	**0.009**	**2.3 (1.2–4.3)**	**0.02**	**2.1 (1.1–4.0)**	**0.03**	**2.4 (1.1–5.2)**	0.07	2.1 (0.9–4.7)	0.07	1.9 (0.9–4.2)	0.1	1.7 (0.8–3.7)
9–19 days	40	**<0.001**	**3.0 (1.7–5.6)**	**0.001**	**2.9 (1.6–5.4)**	**0.001**	**3.4 (1.6–7.2)**	**0.008**	**2.9 (1.3–6.2)**	**0.001**	**3.1 (1.5–6.2)**	**0.008**	**2.6 (1.3–5.4)**
> 19 days	40	**0.01**	**2.2 (1.7–4.1)**	**0.01**	**2.2 (1.2–4.1)**	**0.003**	**3.1 (1.5–6.7)**	**0.004**	**3.1 (1.4–6.7)**	**0.007**	**2.7 (1.3–5.4)**	**0.01**	2.6 (1.3–5.4)

°adjusted to FIGO tumor stage (I vs. II vs. III vs. IV), histological grading (G1 vs. G2 vs. G3), and patients’ median age (≤ 58 vs. > 58 years).

CI—Confidence Interval; HR—Hazard Ratio; Ref—reference

When postoperative residual disease was added to the multivariable survival analyses only residual disease (< 5mm vs. 5–9mm vs. ≥ 10mm) and FIGO tumor stage remained significantly associated with PFS, CSS and OS (data not shown). However, these analyses comprised a subgroup of 108 patients only, who had data on postoperative residual disease available.

In order to assess if the presence of a medical condition for delay of chemo-duration had an impact on survival outcome we compared patients with an underlying medical condition and those without in the subgroup of patients with a delay of at least nine days. We thereby did not find differences between the two groups for PFS, CSS and OS (p = 0.7, p = 0.9, p = 0.7, respectively).

## Discussion

The present study evaluates the impact of the duration of an adjuvant intravenous chemotherapy on the prognosis of patients with epithelial ovarian, peritoneal or fallopian tube cancer. Our data reveal an association between a prolonged duration of chemotherapy and a shortened progression-free, cancer-specific, and overall survival. A delay of total chemo-duration of 9 days or more is thereby associated with a significantly higher risk of progression, of dying from EOC, and of dying from EOC or any other cause, even after adjustment to other prognostic parameters. Moreover, even a short-term delay seems to already negatively affect PFS. To our knowledge this is the first study showing a prolonged duration of adjuvant chemotherapy to be independently associated with PFS, CSS and OS in patients with EOC.

The current mainstay of treatment of patients with EOC is surgical resection followed by six cycles of an adjuvant chemotherapy combining a platinum (carboplatin or cisplatin) and a taxan (paclitaxel or docetaxel) agent. Due to side effects or organizational reasons chemotherapy cycles are often delayed leading to a prolongation of treatment duration. In patients with breast cancer and lymphoma a prolongation of duration and dose reduction of adjuvant chemotherapy are known to have an adverse effect on patients’ survival [15, 16]. In patients with EOC data are controversial. Several studies have been published, describing a RDI of less than 70% or less than 85% to be associated with shorter PFS [10–12]. A recently published Australian study (n = 351) thereby revealed a reduced RDI of <70% to be associated with not only PFS but also OS, even after adjustment to the effects of other prognostic parameters [12].

Only one retrospective study (n = 157) investigated the effect of a delay of chemo-duration separately from dose reduction on patients’ survival [13]. In contrast to our results, this study could not find an association between delay of chemo-duration and OS. We assume this inconsistency is due to differences in treatment and the patients’ collective itself. By comparing the survival curves of patients without delay of chemo-duration, 2-year PFS was 79.6% in our study compared to 20% in the study by Nagel et al. This reflects the fact that only patients with FIGO stages III and IV were included in the study by Nagel et al. However, the majority of patients in our study (68.6%) presented with advanced stage EOC as well. Furthermore, the effects of the delay of chemo-duration remained significant even after adjustment to the effects of FIGO stages, inter alia.

The optimal dose and schedule of chemotherapy in patients with EOC is not yet fully clarified. Recently published prospective trials tried to answer the question on a possible advantage of dose dense chemotherapy [17, 18]. A Japanese study thereby revealed a prolongation of progression free and overall survival after dose dense chemotherapy compared to a three-weekly regimen [17]. Stratification subgroup analyses in the Japanese study showed that the greatest benefit was achieved in patients with residual disease of 1cm or more and who had serous or other histology (not clear-cell or mucinous). Does this indicate that it is primarily the group of sub-optimally operated patients that could eventually benefit or take a disadvantage of variations in adjuvant treatment protocol? Furthermore population-based differences and genetic polymorphisms seem to play a role in pharmacokinetics and effectiveness of chemotherapeutic agents [18, 19].

By comparing chemotherapy-duration to clinico-pathological parameters we could show that the presence of at least 5mm residual disease is associated with a prolonged duration of chemotherapy. This indirectly reflects the patients’ general well-being’s influence on the feasibility of adjuvant chemotherapy. It is yet unclear whether a shorter chemo-duration is only a surrogate parameter for better surgical outcome or if shorter gaps between chemotherapy cycles themselves influence the patients’ prognosis. Furthermore, we found chemo-duration to be significantly prolonged in patients older than 58 years of age when compared to younger patients. This might reflect difficulties for the administration of chemotherapy in the elderly cancer patient [20].

Interestingly, not even one third of the patients received adjuvant chemotherapy in the optimal time frame. The main reasons for treatment delays were hematologic toxicities, followed by postoperative complications associated to the primary cytoreductive surgery. Assessment of risk factors for hematologic toxicities such as neutropenia should be performed in every patient prior to chemotherapy. In selected patients at high risk of neutropenia a prophylactic treatment with a granulocyte colony stimulating factor (G-CSF) should be considered according to international recommendations [21]. Yet, for nearly half of the patients, who had delay, the reasons were not assessable. Organizational issues and individual patient’s wish might be assumed in these cases. Based on the results of the present study, a recommendation for a more diligent scheduling of the respective chemotherapy cycle is warranted.

In order to rule out the possibility of delay of chemo-duration being a surrogate marker for the underlying medical condition we compared survival outcome between patients with and those without known underlying medical conditions. We did not find a difference in survival between the two groups. However, the results of our data cannot exclude a possible independent effect on the patients’ survival outcome of an individual medical condition. As characteristic for a retrospective design our study has several shortcomings, such as lack of random assignment, patient selection, and incomplete data acquisition. We did not take dose adjustment into consideration. Yet, only in one patient a dose reduction was annotated. By excluding all patients who received any other treatment than primary surgery followed by six completed cycles of adjuvant chemotherapy with a combination of platinum and taxan, we have possibly created a selection bias. However, the homogeneity achieved adds an important qualitative component to our study, and fairly compensates for shortcomings in considering dose intensity.

In addition, thanks to our institution’s standardized follow-up program the present study benefits from a long follow-up period of a median of 54 months and an accurate evaluation of progression and cancer-specific mortality.

In conclusion, the present study suggests a prolonged duration of adjuvant chemotherapy after primary surgery to adversely affect PFS, CSS and OS in patients with EOC. According to our results, a delay of the total duration of adjuvant chemotherapy of more than 9 days should be avoided whenever possible. We thereby contribute to the relatively small amount of literature on the role of duration of chemotherapy in patients with EOC available. Yet larger studies are required to validate our results.

## References

[1] Ferlay J, Soerjomataram I, Ervik M, Dikshit R, Eser S, Mathers C, et al. GLOBOCAN 2012 v1.0, Cancer Incidence and Mortality Worldwide: IARC CancerBase No. 11 [Internet]. Lyon, France: International Agency for Research on Cancer; 2013. http://globocan.iarc.fr, accessed on 01/08/2016.

[2] Bristow RE, Tomacruz RS, Armstrong DK, Trimble EL, Montz FJ. Survival effect of maximal cytoreductive surgery for advanced ovarian carcinoma during the platinum era: a meta-analysis. J Clin Oncol 2002;20(5):1248–59. 10.1200/jco.2002.20.5.1248 11870167

[3] Ozols RF, Bundy BN, Greer BE, Fowler JM, Clarke-Pearson D, Burger RA, et al Phase III trial of carboplatin and paclitaxel compared with cisplatin and paclitaxel in patients with optimally resected stage III ovarian cancer: a Gynecologic Oncology Group Study. J Clin Oncol 2003;21(17):3194–200. 10.1200/JCO.2003.02.153 12860964

[4] McGuire WP, Markman M. Primary ovarian cancer chemotherapy: current standards of care. Br J Cancer 2003;89(Suppl.3):S3–8. 10.1038/sj.bjc.6601494 PMC2750616 14661040

[5] Pecorelli S, Favalli G, Gadducci A, Katsaros D, Panici PB, Carpi A, et al Phase III trial of observation versus six courses of paclitaxel in patients with advanced epithelial ovarian cancer in complete response after six courses of paclitaxel/platinum-based chemotherapy: final results of the After-6 Protocol 1. J Clin Oncol 2009;27(28):4642–8. 10.1200/JCO.2009.21.9691 19704064

[6] Du Bois A, Reuss A, Pujade-Lauraine E, Harter P, Ray-Coquard I, Pfisterer J. Role of surgical outcome as prognostic factor in advanced epithelial ovarian cancer: a combined exploratory analysis of 3 prospectively randomized phase 3 multicenter trials: by the Arbeitsgemeinschaft Gynaekologische Onkologie Studiengruppe Ovarialkarzinom (AGO-OVAR) and the Group d’Investigateurs Nationaux Pour les Etudes des Cancers de l’Ovaire (GINECO). Cancer 2009;115(6):1234–44. 10.1002/cncr.24149 19189349

[7] Ben-Josef E, Moughan J, Ajani JA, Flam M, Gunderson L, Pollock J, et al Impact of overall treatment time on survival and local control in patients with anal cancer: a pooled data analysis of Radiation Therapy Oncology Group trials 87–04 and 98–11. J Clin Oncol 2010;28(34):5061–6. 10.1200/JCO.2010.29.1351 20956625 PMC3018356

[8] Chen SW, Liang JA, Yang SN, Ko HL, Lin FJ. The adverse effect of treatment prolongation in cervical cancer by high-dose-rate intracavitary brachytherapy. Radiother Oncol 2003; 67(1):69–76. 12758242 10.1016/s0167-8140(02)00439-5

[9] Repetto L, Pace M, Mammoliti S, Bruzzone M, Chiara S, Oliva C, et al The impact of received dose intensity on the outcome of advanced ovarian cancer. Eur J Cancer 1993;29A:181–4. 8422279 10.1016/0959-8049(93)90169-g

[10] Fauci JM, Whitworth JM, Schneider KE, Subramaniam A, Zhang B, Frederick PJ, et al Prognostic significance of the relative dose intensity of chemotherapy in primary treatment of epithelial ovarian cancer. Gynecologic Oncology 2011;122(3):532–5. 10.1016/j.ygyno.2011.05.023 21658751

[11] Hanna RK, Poniewierski MS, Laskey RA, Lopez MA, Shafer A, Van Le L, et al Predictors of reduced relative dose intensity and its relationship to mortality in women receiving multi-agent chemotherapy for epithelial ovarian cancer. Gynecol Oncol 2013;129:74–80. 10.1016/j.ygyno.2012.12.017 23262376

[12] Anuradha S, Donovan PJ, Webb PM, Brand AH, Goh J, Friedlander M, et al Variations in adjuvant chemotherapy and survival in women with epithelial ovarian cancer—a population-based study. Acta Oncol 2016;55(2):226–33. 10.3109/0284186X.2015.1054950 26079434

[13] Nagel CI, Backes FJ, Hade EM, Cohn DE, Eisenhauer EL, O’Malley DM, et al Effect of chemotherapy delays and dose reductions on progression free and overall survival in the treatment of epithelial ovarian cancer. Gynecologic Oncology 2012;124(2):221–4. 10.1016/j.ygyno.2011.10.003 22055764 PMC4035808

[14] Benedet JL, Bender H, Jones H 3rd, Ngan HY, Pecorelli S. FIGO staging classifications and clinical practice guidelines in the management of gynecologic cancers. FIGO Committee on Gynecologic Oncology. Int J Gyaecol Obstet 2000;70(2):209–62. 11041682

[15] Chang J. Chemotherapy dose reduction and delay in clinical practice: evaluating the risk to patient outcome in adjuvant chemotherapy for breast cancer. Eur J Cancer 2000;36:S11–4. 10785604 10.1016/s0959-8049(99)00259-2

[16] Citron ML, Berry DA, Cirrincione C, Hudis C, Winer EP, Gradishar WJ, et al Randomized trial of dose-dense versus conventionally scheduled and sequential versus concurrent combination chemotherapy as postoperative adjuvant treatment of node-positive primary breast cancer: first report of intergroup trial C9741/Cancer and Leukemia Group B Trial 9741. J Clin Oncol 2003;21(8):1431–9. 10.1200/JCO.2003.09.081 12668651

[17] Katsumata N, Yasuda M, Isonishi S, Takahashi F, Michimae H, Kimura E, et al Long-term results of dose-dense paclitaxel and carboplatin versus conventional paclitaxel and carboplatin for treatment of advanced epithelial ovarian, fallopian tube, or primary peritoneal cancer (JGOG 3016): a randomised, controlled, open-label trial. Lancet Oncol 2013;14(10):1020–6. 10.1016/S1470-2045(13)70363-2 23948349

[18] Pignata S, Scambia G, Katsaros D, Gallo C, Pujade-Lauraine E, et al Carboplatin plus paclitaxel once a week versus every 3 weeks in patients with advanced ovarian cancer (MITO-7): a randomised, multicentre, open-label, phase-3-trial. Lancet Oncol 2014;15(4):396–405. 10.1016/S1470-2045(14)70049-X 24582486

[19] Ma BB, Hui EP, Mok TS. Population-based differences in treatment outcome following anticancer drug therapies. Lancet Oncol 2010;11(1):75–84. 10.1016/S1470-2045(09)70160-3 20129130

[20] Gibson SJ, Fleming GF, Temkin SM, Chase DM. The application and outcome of standard of care treatment in elderly women with ovarian cancer: a literature review over the last 10 years. Front Oncol 2016; 24(6):63. 10.3389/fonc.2016.00063 PMC4805611 27047797

[21] Smith TJ, Bohlke K, Lyman GH, Carson KR, Crawford J, Cross SJ, et al Recommendations for the use of WBC growth factors: American Society of Clinical Oncology Clinical Practice Guideline Update. J Clin Oncol 2015;33(28):3199–212. 10.1200/JCO.2015.62.3488 26169616

